# Medical and Philosophical Intelligence

**Published:** 1817-02

**Authors:** 


### MEDICAL AND PHILOSOPHICAL INTELLIGENCE.

*ROYAL SOCIETY.*—Mr. HATCHETT communicated a process for sweetening musty corn, in a letter to Sir Joseph Banks. Several years ago, this philosopher was engaged in researches into the quality and products of wheat and barley, in consequence of which he discovered that musty grain, which was so bitter as to be totally unfit for use, and which could scarcely be ground, might be rendered perfectly sweet and sound by simply immersing it in boiling water, and letting it remain till the water became cold. The quantity of water in this case was always double that of the corn to be purified. Mr. H. found that the musty quality rarely penetrated through the husk of the wheat; and that, in the very worst cases, it did not extend through the amylaceous matter which lies immediately under the skin. In the hot water, all the decayed or rotten grain swims on the surface, so that the remaining wheat is effectually cleaned from all impurities, and this, too, without any material loss. The wheat is afterwards to be dried, stirring it occasionally on the kiln, when it will be found improved to an extent which can scarcely be believed without actual experience. The immense quantities of musty corn now in merchants' warehouses or granaries belonging to farmers, render these experiments highly valuable at the present crisis; and it is not doubted that, whatever may be the cavils of ignorance and inhumanity, the good sense of all those interested will lead them to adopt so easy, cheap, and effectual a means of rendering their wheat or other grain from 10s. to 40s. a quarter more valuable.

Dec. 12.—The President, happily, was again able to resume his chair, after a more severe return of his complaint than he has experienced these two years.

Proceedings

***Proceedings of the Society of Professors of the Faculty of Medicine at Paris.***

*Curious Case of the general Transformation of the Thoracic and Abdominal Viscera;* presented to the Faculty of Medicine of Paris at the Sittings of Thursday the 12th of December, 1816, by Dr. Beclard, one of the Members, and Chef des travaux Anatomiques de la Faculté de Medicine.—The subject exhibited to the Faculty was a woman aged about 50: the thoracic and abdominal viscera were generally changed from left to right, and *vice versa.* Thus, the right lobe of the lungs, the venæ cavæ and their sinus, the vena agygos, the brachio-cephalic artery, the liver, the pylorus, the duodenum, the end of the mesentery, the cœcum, &c. were on the left side; while the point of the heart, the left lobe of the lungs, the great subclavian, the thoracic duct, the aorta, the spleen, the large extremity of the stomach, the pancreas, the beginning of the mesentery, the sygmoid flexure of the colon, &c. were on the right side, &c. &c. The bottom of the uterus was inclined to the right, as is the ordinary case; and the beginning of the rectum to the left, as is most frequent. The lateral flexion of the dorsal region of the spine was, as usual, concave on the left and convex on the right. The diameter of the axillary artery, and the size of the arm of the same side, were larger than the artery and member of the opposite side.—This is the third case, of the same kind, which M. Beclard has observed in the course of his practice. He concludes, from these facts and several others, relative to the direction of the spine,—1. That there are primitive or original malconformations. 2. That the general transposition of the viscera from right to left, and *vice versa*, is perfectly compatible with life and health. 3. That it requires great care, in exploring the disorders of the breast, not to take a case of transposition for a disorder of the left side of the breast, which had driven the mediastinum to the right. 4. That the transposition in question exists in perhaps the proportion of 1 to 4 or 5000 individuals. 5. That the predominance of nutrition and ordinary action of the right arm does not depend exclusively on the fact of its receiving more directly the blood of the aorta. 6. That the ordinary lateral flexion of the spine does not depend on the pressure of the curve of the aorta, but very probably on the preponderance of the quantity of action of one arm over that of the other. 7. That extraordinary lateral flexions, as those of the hunch-backed, depend on the same cause, or on an inequality in the length of the lower members, acting on the soft bones.

M. le Roux read a Memoir on the Cure of Paralysis by *Nux Vomica.* A conversation arose, in which the Baron Larrey doubted the fact of paralysis in the case in question; and the opinion of most of the members seemed to be, that it was only a slight paralytic affection at the most.

Another Memoir was read, on the quantity of Corrosive Sublimate taken by a Man, the effect of which were transferred to his wife, who died after him in the hospital.

The

The Baron Larrey read the commencement of a Memoir on the Cures of Coxalgie and Rachalgie, as he denominates them, viz. the spontaneous luxation of the os cocygis and the caries of the spine, by the application of Moxa. He exhibited drawings of various cases, and a patient at this moment under his hands, but nearly cured. The caries in the spine has lowered his stature two inches: three of the vertebræ were carious; and the number of applications of moxa found indispensable to triumph over the disorder, hitherto supposed mortal, was twenty-seven, of which we counted the scars. The patient appeared much reduced, and, at present, cannot stoop forward or move the vertebræ without excessive pain; but the caries is completely destroyed, and the action of the vertebræ will, in time, be resumed without pain.*

[We cannot help expressing our surprise at this expectation. It has been ascertained in London, that, whenever a cure takes place in carious spine, it is by anchilososis, and the spine in that part remains inflexible for life.—Edit.]

---

*Central Committee of Vaccine at Paris.*—The annual sittings of the Committee was held at the Faculty of Medicine, on the 2d of January, 1817, under the Presidency of M. Becquey, Under Secretary of the State Department of the Interior—Honorary President the Duke de la Rochefoucauld. After a discourse of the Under Secretary of State, on the object of the sittings, Dr. Judelot, the President in ordinary, and Dr. Husson, Perpetual Secretary, made a Report on the state of Vaccine in France for the year 1816, of which the following is an extract:—

"Notwithstanding the calamities of every kind which have weighed upon France in this disastrous year, the zeal for the propagation of the vaccine has not cooled. Every day the people of the capital, and those of the provinces, familiarize themselves still more with this salutary practice. Every where vaccination is practised gratuitously. There is scarcely a village which does not profit daily of this happy preservative. Individuals rival each other in zeal to administer it. Physicians and zealous surgeons, and even persons unacquainted with the art of healing, magistrates, advise and recommend the vaccine, encourage or practise it themselves, with an assiduity worthy of the highest praise.

"In seventy-two departments of which we have received the returns, the number of vaccinations, in spite of the numerous obstacles occasioned by events, has exceeded one-third of the births; that is, of 626,641 children, born within the year, there have been vaccinated 251,116.

"The measures adopted by the first authorities, in the departments, have powerfully concurred to the success that has been ob-

---

* The Baron Larrey has promised us an extract of his Memoir, for the Medical Journal, which we hope to give in our next.

tained, tained, and are of a nature to produce still greater in happier circumstances. Several Prefects have ordered lists to be made out of all those who have not had the small-pox, and have not been vaccinated: they have obliged the nurses of foundlings to present a certificate of vaccination of the children, before they can receive them. Similar obligations have been imposed on the heads of seminaries. Prisons, and houses of detention, are subjected to the same rules; and the execution is placed under the responsibility of the keepers, or those charged to superintend them. The *sisters of charity*, midwife pupils, are commanded to learn the practice of the vaccine; and the *offices of benevolence*, in the distribution of the public succours, have particular regard to this grand object of all the friends of humanity.

"In the case that the small-pox manifests itself in any place, the parents, or all persons responsible, are bound to make a declaration of the same within three days, in order that efficacious methods may be taken to prevent contagion; and convalescent individuals cannot leave their houses without the certificate of a physician, attesting that there exists no longer any danger of contagion.

"Independent of the prizes, medals, and rewards given annually by government, several prefects have bestowed premiums to persons who have displayed the most zeal in seconding their efforts towards this part of the public good."

At the end of the sittings, his Excellency the Minister of the Interior, represented by the Under Secretary of State, M. Becquey, distributed the prizes instituted in favour of persons who have inoculated the greatest numbers, and who have obtained the greatest success in the propagation of vaccine in 1815, in the following order, viz.

The first grand prize, of the value of 3000 francs (120 guineas), divided between M. Charret, physician at Bourges, who had vaccinated within the year 3,512, and M. Rainaud, physician at Montaubon, who had vaccinated 2018.

The two second prizes, of 2000 francs each, divided between—1, M. Salles, of Valogne, who had vaccinated 2002; and M. Messant, of Aigurande, 1893. 2, M. Serrieres, of Nancy, who had vaccinated 1644; and M. Barry, of Besançon, who had performed 1535 vaccinations.

The three other prizes, of 1000 francs each, were divided between—1, M. le Comte de Neufchateau, 1473; and M. Cazals, of Agde, 1466. 2, Messieurs Giot-Dupré, of Rouen, 1427; and Menard, of Lunel, 1222. 3, Messrs. Courbassier, of Dagnols, 1105; and Noel, of La Chatre, 1048.

Besides these premiums, were given 100 medals of encouragement to as many persons, who had displayed considerable zeal, and vaccinated under 1000 each.

With the above account we are informed of a Mr. Charles Dunne, who asserts that he is appointed by the Vaccine Committee of England, with an establishment of 200l. per annum, to vaccinate in France. It is of high importance to the honour of the English Vaccine Committee, to wipe off such a stain. The French have no need of English vaccinators; and it cannot be believed that the English Vaccine Committee would offer such an insult to the French nation and French physicians. We hope that this man has not improperly used the name of the Committee, which, it is trusted, will, in its own vindication, make known its instructions to Mr. Dunne, and in what manner they were obtained.

[We wish to know what Vaccine Committee our Parisian correspondent refers to.—Edit.]

---

We are glad to find the subject of Craneology brought to that tribunal at which it is likely to be fairly canvassed. Dr. Spurzheim's class increased so much in the northern metropolis, that the Edinburgh reviewer, with a proper sense of decency, felt ashamed of contending on such unequal ground—himself anonymously, and Spurzheim with his name. He therefore required of Dr. Gordon to defend himself in his own character. This has produced the book we announced in our last number; to which, we are informed, Spurzheim has already printed an answer. The former we have received, and, when the latter arrives, shall compare the two, and give the result to our readers. At the same time we conceive it as a duty the Edinburgh reviewer owes to the public and to Spurzheim, that the same publicity should be given to the defence of the German professor as the attack against him received, and in the same publication.

---

*Improved Method of making Bread.*—Mr. Edmund Davy, of the Cork Institution, has communicated the following important facts to the public in The Munster Farmer's Magazine.

"The carbonate of megnesia of the shops, when well mixed with the new flour, in the proportion of from twenty to forty grains to a pound of flour, materially improves it for the purpose of making bread. Loaves made with the addition of the carbonate of magnesia rise well in the oven; and, after being baked, the bread is light and spongy, has a good taste, and keeps well. In cases when the new flour is of indifferent quality, from twenty to thirty grains of the carbonate of magnesia to a pound of the flour will considerably improve the bread. When the flour is of the worst quality, forty grains to a pound of flour seem necessary to produce the same effect.

"As the improvement in the bread from the new flour depends upon the carbonate of magnesia, it is necessary that care should be taken to mix intimately with the flour, previous to the making of the dough.

"I have made a great number of comparative experiments with other substances, mixed in different proportions with the new flour. The fixed alkalies, both in their pure and carbonated state, when used in small quantity, to a certain extent improve the bread from the the new flour: but I have found no substance so efficacious in this respect as the carbonate of magnesia.

"The greater number of my experiments have been performed on the worst new seconds flour I could procure. I have also made some trials on seconds and firsts of different quality. In some cases the results have been more striking and satisfactory than in others—but in every instance the improvement of the bread, by the carbonate of magnesia, has been obvious. It may be necessary to remark, that, in all my experiments, a proper attention was paid to all the circumstances connected with the accuracy of the results. The materials were weighed and mixed together, the dough was made up, and the bread baked, under my own inspection.

"The trials I have made have been necessarily on a small scale; they have been performed chiefly on quantities of flour not exceeding a pound in weight. I shall state the results of a comparative trial on the worst new seconds I could get, with and without the addition of the carbonate of magnesia.

"I made five small loaves, each containing one pound of flour, one hundred grains of common salt, and a large table-spoonful of yeast. The dough, in all of them, was made up with water at the temperature of 100 degrees Farenheit, and exposed before the fire for two hours at the temperature of 70 degrees, to ferment.

The first loaf contained no other addition.

| | | | | |
|---|---|---|---|---|
| The second | 10 | grains of | carbonate of | magnesia. |
| The third | 20 | grains | do. | do. |
| The fourth | 30 | grains | do. | do. |
| The fifth | 40 | grains | do. | do. |

"After they had all been baked together in the same oven, the loaves when cold were examined.

"The loaf without the carbonate of magnesia had fallen in the oven; it was like a cake, and was soft and clammy, and readily adhered to the knife.

"The loaf with the addition of ten grains of the carbonate of magnesia was improved; it had risen better than that which contained none, but still the improvement was not very considerable.

"The loaf with twenty grains was far superior to the one with ten grains; it was for the most part light and porous; but still there was a slight tendency to heaviness. The loaf with thirty grains was still better than the one with twenty grains. But the loaf with 40 grains was uniformly light and spongy, of a more regular texture and better colour than any of the others.

"In all my experiments I have used the porter yeast, but a successful trial has been made with the new barm. I have never made any secret of the foregoing facts, but have readily communicated them to a number of gentlemen, to whom I have shown specimens of the bread made with, and without, the carbonate of magnesia, and they have uniformly given their assent to this improvement.

"I understand a number in Cork, and in the neighbourhood, have have been induced to repeat my experiments; and they have, for the most part, been attended with great success. If there should be any instances of failure, it would not be proper hastily to refer them to a defect in the method, but to impute them rather to a want of attention to those circumstances necessary to insure success.

"I conceive not the slightest danger can be apprehended from the use of such an innocent substance, as the carbonate of magnesia, in such small proportion as is necessary to improve bread from the new flour. It is well known to be administered with perfect safety, even to infants. In order to try the effect of bread made with this substance on myself, I have used it exclusively for the last five weeks, without the least inconvenience, in the proportion of sixty, eighty, and even one hundred grains, to a pound of flour.

"A pound of carbonate of magnesia would be sufficient to mix with two hundred and fifty-six pounds of the new flour, at the rate of thirty grains to the pound. And, supposing a pound of carbonate of magnesia to cost half-a-crown, the additional expense would be only half a farthing in the pound of flour.

"I am not quite satisfied as to the peculiar agency of the carbonate of magnesia, in correcting the bad quality of new flour. It is an extremely light substance, and may probably tend to improve the texture of the bread. The new flour, too, in the process of baking, may, perhaps, be disposed to undergo the acetous fermentation, and the carbonate of magnesia, being slightly alkaline, may correct the tendency of the fermented dough to acidity. On this part of the subject, however, the experiments I am now pursuing will, I trust, throw some light."

---

M. Virey, in a communication to the French Academy of Sciences, (formerly the Institute,) states, that the spur of the rye is not a *champignon* of the genus *Scleroticum*, as M. Decandolle had endeavoured to prove; but, that it is a real disease of the grain; since there are to be found in it all the peculiarities of organization of the rye, a degeneration as yet unknown in its nature, amylaceous fecula, and, probably, all the immediate materials of the Cerealia.

---

*Lizards found in a Chalk Rock.*—Dr. Wilkinson lately presented to the Bath Philosophical Society, a letter he had received from a clergyman in Suffolk, relative to two lizards being discovered by the reverend gentleman in a chalk rock, with some interesting circumstances tending to explain why all the animals which have been discovered in rocks, marbles, &c. die on their exposure to the atmosphere. From observations made, there appeared to be some obstruction in their respiratory organs. One being placed in water disengaged itself from this obstruction; while the other died, from not being enabled to liberate itself from the viscous matter lining the throat. The clergyman in his letter says:

"A pit

"A pit having been opened in the summer of 1814, at Elden, Suffolk, for the purpose of raising chalk, I deemed it a favourable opportunity for procuring fossils; accordingly commissioned the men employed to search for and reserve whatever appeared curious. In this search, I sometimes assisted, and had the good fortune to be present at the discovery of two lizards imbedded in the solid chalk, fifty feet below the surface. The following is the result of my observations;—So completely devoid of life did the lizards appear on their first exposure to the air, that I actually considered them in a fossil state; judge then of my surprise, when, on my attempting to take them up, I perceived them move! I immediately placed them in the sun, the heat of which soon restored them to animation. In this state I carried them home, and immersed one in water, keeping the other in a dry place. You may, perhaps, consider it worthy your observation, that the mouths of the lizards were closed up with a glutinous substance. This obstruction seemed to cause them great inconvenience, which was evident from the agitation perceptible in their throats, and from the frequent distension of the jaws, or rather around the jaws and the head; indeed, they seemed in a state little short of suffocation. The newt which had been immersed in water, after many violent struggles, was at length enabled to open its mouth: this afforded it instant relief, and it evidently derived much satisfaction and comfort from its new element. The other lizard, notwithstanding its repeated endeavours, was unable to open its mouth. It died in the course of the night, probably from being debarred the use of its proper element. The remaining lizard continued alive in the water for several weeks, during which it appeared to increase in size. It disliked confinement; and after many attempts, at length, to my great mortification, effected its escape, nor could I ever after find it."

---

Dr. von Tribolet's (Physician at Bern) *remarkable Observations on the Use of very large Doses of Henbane in Inflammation.* Communicated by Dr. von Embden.—As long ago as the years 1798 and 99, I made use of the hyosciamus as an antispasmodic and narcotic, in internal inflammations, though only in very minute doses; and, according to the directions of my once revered tutor, Professor Richter of Gottingen, only after the depleting indications had been carried to a sufficient extent. Gradually I became more courageous, gave larger doses of it, and found its effects to be purely debilitating, and reducing the activity and morbid irritability of the arterious system. Indeed, at that very time, I considered hyosciamus as a narcotic, but widely differing in its effects from opium; and classed it entirely with the antiphlogistics. I now began using it in pneumonia vera, being rather cautious in the beginning, but becoming more emboldened by degrees; and found the better effects from it the more I increased its dose. In the years 1804 and 5, at a time when sthenical pneumonia was prevalent, I cured many without bleeding, and almost entirely with extract of henbane. The cure was short, and the convalescence quicker than in those patients who, by my colleagues, had been treated with bleeding, and in the usual antiphlogistic method. The following was a most interesting case:—

A robust plethoric servant girl from the country, 22 years of age, caught cold during the period of menstruation, in a dry and cold winter, when the inflammatory fever was epidemical. Menstruation suddenly stopped; a fever, and strong pulmonary inflammation, ensued. Twelve hours after the commencement of the disorder I was called in. Never did I see the indications for blood-letting more strongly marked: terrible congestions in the head and chest were combined with cough and violent dyspnœa. Four scruples of the extract of henbane, dissolved in aqua cerasi, with a little mucilage and antimonial wine, were used during the first twenty-four hours, and one drachm each of the two following days, giving, at the same time, a diluting drink, and applying sinapisms to the feet. All the symptoms soon abated; and the woman recovered in a very short time, without having any bad consequences.

A case of Croup follows, treated with the same success, after which the author proceeds—

The following are the results of my observations on that head:—Henbane is to be used in the commencement of the disorder, before polypous excretions have taken place: after this it becomes hurtful.* It must be given in strong doses, at least two grains every half hour to a child of from two to three years, and be continued till the inflammatory symptoms abate, and the respiration becomes more free, when the dose is to be diminished, and again increased in the exacerbations. It is thus necessary that the physician should see the child every two hours. It entirely supersedes blood-letting, leeches, and calomel. I give nothing with it but the antimonial wine, applying sinapisms and vesicants to the legs.

Dr. Hufeland remarks on the above,—the observations of so high and respectable an authority certainly deserve the greatest attention, for they shew what may be effected with remedies which are still neglected by many physicians, as also the doses in which they may, and, in many cases, must, be given, to answer the purpose. In my practice, (says he,) hyosciamus ever was an important, nay a favourite, remedy, to which I owe the best of my cures; and ever was of great assistance to me in numerous disorders. In all chronic and acute disorders, which are combined with enormously exalted sensibility, it serves as a mediator in pro-

* We are entirely at a loss to know what is here meant by polypous excretions taking place in inflammation. If the author intends effusion of coagulated lymph, such occurred in his case of croup successfully treated.—Edit.

curing curing the remedies, destined to act against the organic anomalies, a freer admission, in rendering them more pleasant to the nerves, in preventing anomalous re-actions; in short, in regulating the whole cure. But more particularly is it adapted for inflammatory disorders, on account of its being the only narcotic which, at the same time that it lowers the sensibility, does not exalt the irritability, and the action of the vascular system; and does not hinder the secretions and excretions, as is the case with opium, which, on that account, always remains a doubtful remedy in inflammatory disorders. Dr. H., however, confesses never to have dared to use it in doses equal to those of Dr. T.

---

A very violent tempest, uniting all that is dreadful contained in the atmosphere, on the 12th of June last, befel the city of Werschetz, in Hungaria. Storm, hail, and electric shocks, contended for the superiority. The terror of the inhabitants was dreadful during this scene; and, from that day to the 13th of July, seven women were delivered of still-born children, in consequence of the mental agitation they had endured. After the hurricane in Madeira about twelve years ago, it was asserted that no woman in Funchall, pregnant at the time, produced a living child at the expected period.

---

### *On Legal and Judicial Chemistry*; by Dr. Chaumeton.

*(Continued from vol. 36, p. 432.)*

I am always greatly surprised to find that amongst these names I do not see that of Chaptal, who has so eminently contributed to the progress of the arts, *consilioque manuque.* It is upwards of twenty years since this learned chemist has published two excellent methods of varnishing pottery. The first consists in dipping the articles in a solution of earth of murviel in water, then drying them, and afterwards dipping them in a solution of green glass (ground very fine) and water: this layer of vitrified power attaching itself to the argil of the murviel, produces a very smooth white cheap varnish or glaze. The second method consists in steeping the dried earthen vessels in a strong solution of marine acid, and afterwards baking them.

Serious dangers, to which they are perpetually obliged to expose themselves, are the melancholy lot of many professions, so that those who exercise them are compelled to walk *inter pericula et mortes*. Painters, gilders, varnishers, &c. live almost constantly in an atmosphere charged with pernicious metallic exhalations. A complexion dyed with the metallic oxides, dreadful cholics, nervous affections, paralysis, and premature death, are too often the only recompense of the ablest workmen.

The police ought to watch over the confectioners with an extreme vigilance, as they ornament their sweetmeats *(bonbons)* with brilliant and poisonous colours. The police ought also to extend its superintendance over tinmen, dyers, toymen, and even card-makers.

Commerce,

Commerce, and the preparations of medicaments, are objects of the highest importance, for the sophistication of a remedy may aggravate the disorder, and even cause the death of the patient. To remedy this, it would be well if government were to appoint a commission of the most eminent physicians, apothecaries, chemists, and druggists, with authority to enter the shops and warehouses of apothecaries, chemists, druggists, &c. and examine the state of all medicaments, and ascertain their coincidence with the formulæ of the Pharmacopœia, and whether time, accident, or the undue admission of atmospheric air, have not deteriorated their qualities, and rendered them unfit for use, as being uncertain in their operation.

In a sketch, as exact as it is concise, the author points out the means chemistry offers for discovering the adulteration of the principal mineral, vegetable, and animal substances used in medicine. He deplores the criminal custom of falsifying musk, which, in a state of purity, would be one of the best therapeutic agents. It would have been greatly to be desired that so competent a judge as Dr. Remer, in speaking of the introduction, or rather the re-introduction, into medicine of nitrate of silver, lead, copper, and arsenic, had better appreciated the just value of such an acquisition.

I have known the Professors of Berlin order the *lapis infernalis* internally for the epilepsy with the same composure as they would have ordered valerian or orange leaves. I have seen, in several parts of Germany, and especially in Wetteravia, distinguished medical men administer acetate of lead in consumption with as much boldness, and more confidence, than I would have ordered asses' milk, or iceland moss. I have seen physicians at Paris prefer arsenic to quinquina, for the cure of intermittent fevers.

These examples have not seduced me, though a most zealous partizan of new discoveries, who consider the most violent poisons as susceptible of being converted by the man of genius into heroic remedies. I cannot even admit, without restriction, the extravagant praises that M. Guersent lavishes on copper. " When we administer preparations of copper, in sufficiently strong doses, at the moment a strong irritation is produced on the organs of digestion, from whence result vomiting and alvine evacuations. But, if slighter doses are given, and continued for some time, a general excitation of the blood-vessels and nervous system is the consequence; and, in fact, a daily use of salts of copper, in doses sufficient to produce evacuations, and, at the same time, increase the action of the solids—these medicaments then act secondarily on the lymphatics as alteratives."

I admire the noble sentiments of Dr. Remer, who, inspired with the dignity of the profession, exclaims, " Perhaps the high idea I have formed of the duties of the physician may transport me too far, but I am firmly persuaded that the moment a medical man has discovered a new means of relieving humanity, the discovery is no longer his private property, but he ought instantly to enable enable all the world to enjoy it. Glory to the virtuous Jenner, who, convinced of the inappreciable advantages of the vaccine inoculation, did not bury under the shade of mystery the fruit of his important researches. Pride of England, and benefactor of human nature, receive my homage. Alas! why has not thy conduct, so worthy of serving as a model, been universally imitated?"

The atmosphere we breathe exercises on the frame a continual and powerful influence, according to the degree of temperature. The meteors which agitate and modify it, the effluvia which alter and corrupt it, M. Remer does not exaggerate; but, as a faithful historian, he enumerates the injuries arising from theatres of anatomy, cemeteries, slaughter-houses, privies, canals, forges, caverns, and other sources of fetid gas and unhealthy miasma. He brands, as a crime against humanity, the ordonnances which re-establish the interment in churches, in Tuscany, in 1803, which had been so wisely suppressed by the Archdukes Leopold and Ferdinand, the predecessors of the imbecile King of Etruria. I may be here permitted to cite the expressions of the Spanish Doctor Ramon Lopez Mateos.* The greater part of sovereigns do not, like the Grecian chief physician, consider the ανηρ πολλων αντaξιος αλλων. It is from having neglected to follow his advice, that the prisons, barracks, and hospitals, become the frightful abodes of pestilence, sinks of corruption, and living tombs. Burial in churches has produced incalculable evils. Is it not offering an insult to the Deity to burn on his altars an incense the perfumes of which are blended with the pestiferous stench of bodies in a state of putrefaction?

A comparative analysis of the various modes of purifying the air in hospitals, &c. and removing infection, displays at once an able philosopher, a learned chemist, and a judicious physician. M. Remer pays a just tribute of praise and gratitude to the memory of the immortal Guyton Morveau, whose loss is universally deplored by every friend of science.

According to the progress of civilization, the wants of society increase, and new sources of enjoyment are continually hunted after, the means of procuring which are sometimes ridiculous, and sometimes dangerous, and even criminal. Amongst those reviewed by the author, I will cite that of snuff, a black dirty powder, which transforms the prettiest face and the most interesting physiognomy into an object of horror and disgust. The custom of paint is as absurd as it is general, or rather it is general, precisely because it is absurd.

It would be infinitely painful, but, at the same time, extremely curious, to see a living man take fire on a sudden, inflame, consume, and be reduced to ashes, before the eyes of an astonished spectator. Before I place entire confidence in such a phenomena, it must be attested by witnesses known to be every way irreproachable.

---

* Pensamientos sobre la razon de las leyes; in 8vo. Madrid, 1810.

After giving a rapid sketch of the falsification of money and titles, letters, diplomas, charts, and documents of every kind, the author enters upon an immense field of enquiry, highly important, but surrounded with difficulties at every step. Thousands of volumes have been published on Poisons, and millions of innocent animals have been tortured and martyred, with scarcely any benefit to science.

M. Remer does not make his reader shudder in displaying with ostentation the list of his victims; he does not boast of having poisoned even a single dog;* and yet what he says of venomous substances appears as profoundly conceived as it is elegantly expressed.

This excellent judicial toxicology, if I may so call it, terminates this extraordinary learned and important work, which merits a distinguished place in the library of the magistrate as well as of the physician. F. P. CHAUMETON.

---

*Medical Bibliography—Winter*, 1816; abridged from Dr. CHAUMETON.

The career I propose to myself is long and painful; and yet it only requires to give a rapid glance on the various productions which daily enrich or burthen medical literature. How many journalists would find this task agreeable and easy to perform. There is nothing more easy, in fact, than to accumulate titles without offering to the reader a judicious reflection, or a rational judgment of the work. My object is not to follow their steps: whenever I render an account of a work, the reader may be assured that it is only after having read it through, and seriously meditated upon it.

I have thought it necessary to say thus much by way of preface to the plan I have deemed it proper to adopt: it may be thought singular; but, if I had not been convinced of its utility, I should have renounced it entirely. It is not subjecting myself, without scruple, to rules as minute as they are superfluous; it is not in confining myself strictly within certain limits, that I shall attain the object I have in view. Let me not be reproached with having forgotten such or such productions, loudly praised by such or such a celebrated journal. Sometimes I shall pass in silence the ponderous quarto, and consider, in preference, the modest unassuming duodecimo. My intention is to collect an abundant

---

* M. Remer, however, does not condemn these poisoners of animals: he declares even that dogs seem to be destined to be the martyrs of science. Heaven preserve me from being of such a dreadful opinion. I admire the dog, because, in all ranks of created beings, he is the only one that offers to my notice an example of all the virtues; and, if to make the most brilliant discovery, it were necessary to torture that faithful and heroically-devoted animal, I would refuse to purchase the glory at such a price.

harvest harvest. Every thing that has appeared in medical literature since the commencement of the 19th century will be open to me:—that limit is, perhaps, the only one I shall engage to observe, as I shall not adopt a chronological order in my researches. The review of every three months will comprise, not all that has been published in the period, but all I may have read; and shall rather consider material facts, than points of doctrine breathing more the spirit of system than the love of science and the diffusion of knowledge.

ANATOMY.

Anatomy is, beyond all dispute, the basis of medicine: it is the point where we ought to commence the honourable (rather than honoured) career of the art of healing. The editing of a work of anatomy, to smooth the rugged paths of science to beginners, is no ordinary labour, which many conceive themselves so competent to perform, that we are inundated with books of anatomy of every kind—as general, descriptive, human, comparative, and pathological. In the midst of this immense collection, majestically soars the two anatomical works of Bichât, which it is enough to name, their merits being so well known and admired.

The *Bibliotheca Anatomica* of Haller is a mine of riches: unfortunately he ceased to write in 1776. The historical sketch of Portal abounds with inconceivable mistakes. *L'Histoire de l'Anatomie*, by Thomas Lauth, is a cold, dry, and heavy performance, like the rest of the works of that author, though it is more correct than the work of the Paris academician.

*Cloquet.*—The minute description of a bone, a muscle, a ligament, a cartilage, or an aponeurosis, is, undoubtedly, highly useful; but it does not furnish any food for genius, and excludes the flowers of rhetoric and the excursions of the imagination. It is a real sacrifice to science;* and the more generous, because M. Cloquet possesses, in an eminent degree, the art of speaking and writing well, as is evident from his thesis,† and his lectures as professor at the Atheneum.

To return to the treatise of Descriptive Anatomy, a Latin dedication at the head of a French work has always appeared to me improper.

M. Cloquet rejects the ordinary divisions of anatomy, to adopt those of Professor Dumeril, which consists in examining the organs in a physiological order, according to their various relations with other bodies, which concur to the nutrition of the individual, or ought to be employed for the propagation of the species. I do not deny the great advantages of this method, professed by the celebrated naturalist: it is certainly more philosophical, and leads

---

* Traite d'Anatomie descriptive redigé d'apres l'ordre adopte a la Faculté de Medicine de Paris. 2 vols. 8vo. Paris, 1816.

† Dissertation sur les Odeurs sur les Sens et les Organes de l'Olfaction. 4to. Paris, 1815.

to more beautiful conclusions than the ordinary method, which, in other respects, appears also to have its advantages; but I wait with impatience the remainder of the work, which is to contain the comparative anatomy of different ages and sexes, and the more prominent points of the anatomy of animals compared with that of man, and the applications of anatomical knowledge to physiology, pathology, and surgery. All these subjects, of high importance, will undoubtedly acquire a new degree of interest by the manner in which they will be treated.

*Pathological Anatomy of Cruveilhier.*—In waiting for the grand and long-promised work of M. Dupuytren, his countryman and pupil has published an *Essai sur l'Anatomie Pathologique en general, et sur les Transformations et Productions Organiques en particulier.* (2 vols. 8vo. Paris, 1816.) The style of the work is very unequal, but that may be referred to the youth of the author, who, full of enthusiasm on his favourite subject, beholds all medical science comprised in pathological anatomy, slighting, in my opinion, too much the undoubted benefits arising from the applications of comparative anatomy.

Amongst his observations is a very singular one, to say the least of it. It is, that Surgeon-Major Broussard declares to have seen, in the retreat from Moscow, an unfortunate wretch who had his eyes frozen, who is still alive. If this be not a mere tale, it is a new proof that the truth may occasionally be extremely improbable.* However this may be, it cannot be denied that the work of M. Cruveilhier displays genuine talent; and, from such a debut of a young author, the greatest hopes are to be entertained.

---

*Blindness cured by Moxa.*—Many English prisoners of war in Spain can bear testimony to the paternal care and exquisite skill of Baron LARREY, first surgeon of the Imperial Guard. We relate the following only on account of its importance in the art of healing. The sick and wounded English prisoners at Valladolid were confided to the care of Baron Larrey; the fatigues, the cold and damps, in crossing the mountains of the Asturias, added to the privations of every kind they had suffered, only preceded the hospital fever; but, by the care of the baron, in removing those not yet afflicted with it to healthy apartments, and using all his skill on the others, it was soon got under; they were, however, almost naked, and the baron solicited and obtained for them great coats, shirts, and shoes, of which they were in the greatest want.

A young drummer, aged twelve or thirteen, the son of a cor-

---

* We see nothing incredible in the above. The eyes might be frost-bitten by the freezing of the secretion with which they were bedewed; nor is it at all improbable that they were the only parts exposed to the atmosphere. After being thus killed, suppuration from the sockets would be an easy process. We may further remark, the effects of radiant cold so beautifully explained by Dr. Wells.—English EDIT.

poral of the 12th light infantry (English), was, with his father, in the number of the prisoners. "This child, (says the Baron) whom the father kept continually on his lap, was perfectly blind; the pupils had scarcely any motion. This blindness, from the account given by the father, came on suddenly in crossing the mountains of the Asturias, during the dreadful season we had just passed. The cold too was likely to have affected him the more sensibly, as he had recently had his hair cut close, and he had travelled from Corogna to Valladolid barefoot, as nearly all the prisoners had done. We easily discovered in this child the formation of the gutta serena, produced by the union of the two causes we have mentioned.

"It would be difficult to paint the situation of the father, and his affliction for the state of his son; and several of his comrades partook of his sorrow, for the boy was much liked in the regiment; indeed, I perceived, with pleasure, that in general the prisoners bore a reciprocal attachment to each other, and exhibited a mutual, tender, and generous affection.

"As the blindness of this child was recent, I conceived the hope of curing him. I ordered him to be brought into one of our hospitals, as well to be more out of the danger of the hospital fever, as to have him more immediately under my own eye. I at first ordered him a soap bath; I then administered diaphoretic bitters, and applied moxa on the facial nerve at its point of emergence from the cranium behind the angle of the jaw; his head was rubbed with a camphorated alkaline liniment, and covered with a flannel cap, and his eyes were rubbed with camphorated hot wine.

"On the second application of moxa, the boy saw the light; at the fourth, he distinguished objects and colours; and, on the seventh, the visual functions were totally re-established. I took care to prevent inflammation and suppuration from the burns occasioned by the moxa, by applying ammonia instantly on the wound. To secure this extraordinary success, I established a cautery in the arm, and procured the young prisoner a little Spanish cloak, in order that he might be more warmly clothed; and, I had the happiness of seeing him depart for France perfectly cured, with his father, who knew not how sufficiently to express his joy.

"This, with numerous other cases, which I have witnessed, proves the great efficacy of moxa in paralysis, and I cannot sufficiently recommend the use of it.—D. J. LARREY."

The baron observed a curious case in another soldier of the same regiment. He was convalescent of a nosocomial fever, when he was suddenly attacked with an anasarca, which confined itself to the right side of the body; the left remaining nearly in the natural state. This second disorder resisted, for a long time, all the efforts of art; but was, at length, conquered by the care and perseverance of the French surgeons.

---

An institution, much wanted in the metropolis, for the Diseases of the Ear, has lately been established,—of which Mr. CURTIS, of Soho-square, is appointed surgeon.

Dr. Adams's Lectures will commence on Tuesday the 4th instant.

Dr. Merriman and Dr. Ley will re-commence their Course of Lectures on the Theory and Practice of Midwifery and the Diseases of Women and Children, at the Middlesex Hospital, on Monday, February 17th, at half-past ten o'clock.

Dr. Davis will commence his next Course of Lectures on the Theory and Practice of Midwifery, and the Diseases of Women and Children, on Thursday the 13th February.

Mr. J. H. Curtis, Surgeon and Aurist, Surgeon to the Royal Dispensary for the Diseases of the Ear, will begin his Course of Lectures on the Anatomy, Physiology, and Pathology, of the Ear, on Thursday the 27th of February, at seven o'clock in the evening, at his house, No. 18, Soho-square.

Mr. Stevenson purposes commencing his annual Course of Lectures on the Anatomy, Physiology, and Pathology of the Eye and Ear, early in March. In a series of twenty-four Lectures will be given a full anatomical description and demonstration of every part of the complex and very delicate structure of the organs of sight and hearing; an explanation of their respective functions in a state of health; and an elucidation of the history, symptoms, and best mode of treating their various diseases: including an account of all the requisite surgical operations, and the most approved methods of performing them.

---

A Physician, having, for the last two years, been making experiments in his kitchen, with a view of composing a Culinary Code for rational Epicures, and augmenting the alimentary enjoyments of Private Families, has nearly completed the work, which will be entitled "Apicius Redivivus, or, The Cook's Oracle;" wherein especially the art of composing soups, sauces, and flavouring essences, will be made so clear and easy, by the quantity of each article being accurately stated by weight and measure, that every one thereby may soon learn to dress a dinner as well as the most experienced cook.

---

Dr. Adams's Life of Mr. Hunter will certainly appear before the anniversary of his birth-day, the 14th instant.

---

In the press, and shortly will be published, in 2 vols. 8vo. embellished with eight engravings, by James Sowerby, F.L.S., a Midland Flora: comprising the indigenous plants of the more central counties; with occasional notes and observations, and a short introduction to the study of Botany. By T. Purton, Surgeon, &c. Alcester, Warwickshire.

---

In the press, and shortly will be published, Some further Observations on the subject of the proper Period for Amputating in Gun-Shot Wounds, accompanied by the official Reports of the Surgeons employed in his Majesty's ships and vessels at the late Battle before Algiers. By A. Copland Hutchison, late Surgeon to the Royal Naval Hospital at Deal.

BILLS

### BILLS OF MORTALITY.

### *Diseases and Casualties of the Year* 1816.

| DISEASES. | |
|---|---|
| Abortive & Stillborn | 734 |
| Abscess | 106 |
| Aged | 1913 |
| Ague | 3 |
| Apoplexy & Suddenly | 434 |
| Asthma | 1003 |
| Bed-ridden | 5 |
| Bile | 1 |
| Bleeding | 30 |
| Bursten & Rupture | 35 |
| Cancer | 79 |
| Chicken-pox | 1 |
| Childbed | 234 |
| Colds | 19 |
| Colic, Gripes, &c. | 6 |
| Consumption | 4272 |
| Convulsions | 3264 |
| Cough and Hooping Cough | 666 |
| Cramp | 2 |
| Croup | 92 |
| Diabetes | 5 |
| Dropsy | 788 |
| Dysentery | 1 |
| Epilepsy | 4 |
| Evil | 8 |
| Fevers of all kinds | 1299 |
| Fistula | 8 |
| Flux | 15 |
| French Pox | 61 |
| Gout | 56 |
| Gravel, Stone, Strangury | 14 |
| Grief | 4 |
| Headmoldshot, Horseshoehead, & Water in the Head | 408 |
| Inflammation | 977 |
| Jaundice | 76 |
| Jaw Locked | 2 |
| Leprosy | 1 |
| Lethargy | 1 |
| Livergrown | 79 |
| Lunatic | 230 |
| Measles | 1106 |
| Miscarriage | 7 |
| Mortification | 327 |
| Palpitation of the Heart | 11 |
| Palsy | 195 |
| Pleurisy | 22 |
| Purples | 2 |
| Quinsy | 2 |
| Rash | 1 |
| Rheumatism | 14 |
| Rising of the Lights | 1 |
| Scrofula | 2 |
| Scurvy | 2 |
| Shingles | 1 |
| Small-pox | 653 |
| Sore Throat | 13 |
| Sores and Ulcers | 15 |
| Spasm | 43 |
| St. Anthony's Fire | 7 |
| Stoppage in the Stomach | 26 |
| St. Vitus's Dance | 1 |
| Swelling | 2 |
| Swine Pox | 1 |
| Teeth | 417 |
| Thrush | 89 |
| Tumor | 3 |
| Water in the Chest | 48 |
| Worms | 15 |

| CASUALTIES. | |
|---|---|
| Broken Limbs | 3 |
| Burnt | 48 |
| Drowned | 105 |
| Excessive Drinking | 13 |
| Executed | 10 |
| Found Dead | 31 |
| Fractured | 4 |
| Frighted | 6 |
| Killed by Falls & several other Accidents | 56 |
| Killed by Fighting | 1 |
| Killed by Swallowing a Shilling | 1 |
| Killed themselves | 50 |
| Murdered | 8 |
| Overlaid | 2 |
| Poisoned | 8 |
| Scalded | 5 |
| Suffocated | 3 |

| | | | |
|---|---|---|---|
| Christened | Males | 12132 | In all 23581 |
| | Females | 11449 | |
| Buried | Males | 10105 | In all 20316 |
| | Females | 10211 | |

Whereof have died,

| | | | |
|---|---|---|---|
| Under Two Years of Age | 5400 | Sixty and Seventy | 1720 |
| Between Two and Five | 1960 | Seventy and Eighty | 1308 |
| Five and Ten | 845 | Eighty and Ninety | 781 |
| Ten and Twenty | 675 | Ninety and a Hundred | 168 |
| Twenty and Thirty | 1464 | A Hundred | 3 |
| Thirty and Forty | 1912 | A Hundred and One | 0 |
| Forty and Fifty | 2123 | A Hundred and Three | 1 |
| Fifty and Sixty | 1955 | A Hundred and Four | 1 |

Increased in the Burials this Year 756.

There have been Executed in the City of London and County of Surrey 25; of which Number 10 only have been reported to be Buried within the Bills of Mortality.

The reader will observe an increase of 756 deaths, a number very inconsiderable. The number of christenings has also increased, creased, by which it would appear, that, notwithstanding the admitted distresses, our population has not lessened. To this remark we should add, that the extensive parishes of Mary-le-bone and St. Pancras, the former equal in population to all Bristol and its environs, and the latter to the whole arch-deaconry of Harford, and both situated in the most healthy part of the town, are not included in the bills. The number of deaths by inflammation is stated at about 20 more than last year. To this list should be added the cases of pleurisy, and, probably, most of the water in the head. The deaths by small-pox are fewer than last year, which proves that the disease has been mild, as all practitioners admit its greater prevalence. The number of deaths by measles is truly formidable, and has been progressively increasing for some years; they exceed the former bill by nearly 400; but how many of them should be put to scarlatina, it is impossible to say. Equally uncertain is the number of that disease, compared with other fevers, as they are now most absurdly all blended in one common article.

One thing worthy of notice is the greater mortality of females above males: the number is, however, not considerable, and, probably, may be accounted for by the number of men who have emigrated, or who are connected with the army and navy.

The complaints of the last two months, like those of the whole year, have been principally those of spontaneous inflammation, arising, as it should appear, from plethora. Hæmorrhage has, in the reporter's observation, been more common, of late, since the weather has been milder. A remarkable case of this kind has occurred in a young lady, only five years old, who had a similar attack (per anum) about the same time last year. The quantity voided was from a table-spoonful to nearly six ounces of apparently arterial blood, by its florid appearance when first voided, and by its firm coagulation. It is in the recollection of the family that this child's grandfather had a similar discharge still earlier in life, and that it continued as long as he lived, at different periods.

The winter asthmas have been particularly mild, and the coughs less troublesome, excepting during a short epidemic, the effects of which have nearly ceased.

A case of chronic inflammation in the brain has occurred in a young gentleman who has just arrived at his full growth. It has proved extremely insidious, but hitherto the faculties do not seem to have suffered; the senses are somewhat more acute than natural. Another, nearly similar, has occurred over the left eye of a female somewhat advanced in life.

### METEOROLOGICAL REGISTER.

*From December the 25th, 1816, to January the 25th, 1817.*

Kept by C. BLUNT, Philosophical Instrument Maker, No. 38, Tavistock-Street, Covent-Garden.

| Moon. | Day. | Wind. | Barometrical Pressure. Max. | Min. | Mean. | Temperature. Max. | Min. | Mean. | |
|---|---|---|---|---|---|---|---|---|---|
| ◑ | 26 | SW | 29·82 | 29·70 | 29·76 | 35 | 26 | 30· | Rain |
| | 27 | SW | 29·40 | 29·36 | 29·28 | 31 | 24 | 22· | Rain |
| | 28 | SW | 29 60 | 29·56 | 29 58 | 30 | 18 | 24· | Fair |
| | 29 | SW | 29·72 | 29·70 | 29·71 | 46 | 39 | 42· | Rain |
| | 30 | SW | 29·99 | 29·93 | 29·96 | 48 | 40 | 44· | Rain |
| | 31 | SW | 29 80 | 29·80 | 29·80 | 48 | 21 | 31· | Rain |
| | 1 | W | 29·60 | 29·60 | 29 60 | 41 | 24 | 32· | Rain |
| | 2 | SW | 29·42 | 29·40 | 29·41 | 50 | 39 | 44· | Rain |
| ○ | 3 | SW | 29·44 | 29 40 | 29·42 | 46 | 36 | 41· | Rain |
| | 4 | S | 29·25 | 29·21 | 29·23 | 44 | 38 | 36· | Rain |
| | 5 | NW | 29 41 | 29·40 | 29.40 | 43 | 36 | 39· | Rain & Snow |
| | 6 | NW | 29·69 | 29·63 | 29·66 | 44 | 33 | 38· | Rain |
| | 7 | NW | 30 44 | 30 40 | 30·42 | 42 | 24 | 33· | Fair |
| | 8 | NE | 30·35 | 30 25 | 30·30 | 39 | 29 | 34· | Fair |
| | 9 | NE | 30 66 | 30·60 | 30·63 | 39 | 26 | 32· | Fair |
| ◐ | 10 | N | 30·71 | 30·69 | 30·70 | 40 | 33 | 36· | Frost |
| | 11 | NW | 30·52 | 30·50 | 30·51 | 37 | 33 | 35· | Frost |
| | 12 | NW | 30·19 | 30 17 | 30·18 | 38 | 34 | 32· | Frost |
| | 13 | W | 29·85 | 29·81 | 29·83 | 38 | 30 | 34· | Rain |
| | 14 | NW | 29·61 | 29·60 | 29·60 | 32 | 25 | 28· | Fair |
| | 15 | NW | 28 96 | 28 94 | 28·95 | 45 | 37 | 41· | Snow |
| | 16 | NW | 29·38 | 29·32 | 29·35 | 46 | 36 | 41· | Rain |
| ● | 17 | NW | 28·99 | 28·99 | 28·99 | 47 | 35 | 41· | Fair |
| | 18 | W | 29·50 | 29·50 | 29·50 | 53 | 40 | 46· | Fair |
| | 19 | W | 29·17 | 29·13 | 29 15 | 32 | 25 | 28· | Fair |
| | 20 | S | 28·84 | 28·84 | 28·84 | 46 | 36 | 41· | Rain |
| | 21 | SW | 29·83 | 29·83 | 29·83 | 49 | 38 | 44· | Rain |
| | 22 | SW | 29 87 | 29·83 | 29·85 | 53 | 39 | 41· | Fair |
| | 23 | SW | 30·11 | 30·09 | 30·10 | 51 | 41 | 46· | Fair |
| | 24 | SSW | 30 35 | 30 31 | 30·33 | 57 | 42 | 49· | Fair |
| ◑ | 25 | SW | 29·41 | 29·41 | 29·41 | 52 | 40 | 46· | Fair |

RESULTS.

| | | | | | |
|---|---|---|---|---|---|
| Mean barometrical pressure of the month | | 29·98 | Mean temperature of the month | | 37 deg. |
| Maximum 30·71, | wind at | N | Maximum 57, | wind at | SSW |
| Minimum 28·84, | ——— | S | Minimum 18, | ——— | SW |

*Scale exhibiting the prevailing Winds during the Month.*

| N | NE | E | SE | S | SW | W | NW |
|---|---|---|---|---|---|---|---|
| 1 | 2 | 0 | 0 | 2 | 13 | 4 | 9 |

| | Mean barometrical pressure. | Mean temperature. |
|---|---|---|
| From the first quarter on the 26th Dec. to the full moon on the 3d Jan. | 29·64 | 33· |
| ——— the full moon on the 3d, to the last quarter on the 10th | 29·97 | 36· |
| ——— the last quarter on the 10th, to the new moon on the 17th | 29·63 | 36· |
| ——— the new moon on the 17th, to the first quarter on the 25th | 29·50 | 43· |

